# Seizures after decompressive surgery for cerebral venous thrombosis

**DOI:** 10.1007/s00415-025-13410-8

**Published:** 2025-10-10

**Authors:** Mayte Sanchez van Kammen, Sanjit Aaron, Jorge M. Ferreira, Patrícia Canhão, Adriana B. Conforto, Antonio Arauz, Marta Carvalho, Jaime Masjuan, Vijay K. Sharma, Jukka Putaala, Maarten Uyttenboogaart, David J. Werring, Rodrigo Bazan, Sandeep Mohindra, Jochen Weber, Bert A. Coert, Prabhu Kirubakaran, Pankaj Singh, Diana Aguiar de Sousa, Jonathan M. Coutinho, José M. Ferro

**Affiliations:** 1 Department of Neurology, Amsterdam University Medical Centers, University of Amsterdam, AMC, Meibergdreef 9, 1105 AZ Amsterdam, The Netherlands; 2 Neurology Unit, Department of Neurological Sciences, Christian Medical College and Hospital, Vellore, Tamil Nadu India; 3 Serviço de Neurologia, Centro Hospitalar Universitário Lisboa Central, Lisbon, Portugal; 4 Serviço de Neurologia, Departamento de Neurociências E Saúde Mental, Unidade Local de Saúde Santa Maria, Lisbon, Portugal; 5 Centro de Estudos Egas Moniz, Faculdade de Medicina, Universidade de Lisboa, Lisbon, Portugal; 6 Hospital das Clínicas, Universidade de São Paulo, São Paulo, Brazil; 7 Stroke Clinic, Instituto Nacional de Neurología y Neurocirugía, Mexico City, Mexico; 8 Serviço de Neurologia, Departamento de Neurociências Clínicas E Saúde Mental, Unidade Local de Saúde São João, Faculdade de Medicina da Universidade Do Porto, Porto, Portugal; 9 Servicio de Neurología, Departamento de Medicina, Hospital Universitario Ramón y Cajal, IRYCIS, Universidad de Alcalá. Red INVICTUS, Madrid, Spain; 10 Department of Medicine, Yong Loo Lin School of Medicine, National University of Singapore, Singapore, Singapore; 11 Department of Neurology, Helsinki University Hospital and University of Helsinki, Helsinki, Finland; 12 Department of Neurology and Medical Imaging Center University Medical Center Groningen, University of Groningen, Groningen, The Netherlands; 13 Stroke Research Centre, UCL Queen Square Institute of Neurology, London, UK; 14 Faculdade de Medicina Campus de Botucatu, Universidade Estadual Paulista Julio de Mesquita Filho, Botucatu, São Paulo, Brazil; 15 Department of Neurosurgery, Post Graduate Institute of Medical Education and Research (PGIMER), Chandigarh, India; 16 Department of Neurosurgery, Steinenberg Clinic, Reutlingen, Germany; 17 Department of Neurosurgery, Amsterdam University Medical Centers, University of Amsterdam, Amsterdam, The Netherlands; 18 Stroke Center, Centro Hospitalar Universitário Lisboa Central—ULS S José, Lisbon, Portugal

**Keywords:** Intracranial sinus thrombosis, Decompressive craniectomy, Seizures, Prognosis

## Abstract

**Introduction:**

The incidence and outcomes of seizures among patients with severe cerebral venous thrombosis treated with decompressive surgery are unknown as are potential predictors of seizures in these patients.

**Patients and methods:**

We report data from a multicenter, consecutive cohort including patients with cerebral venous thrombosis treated with decompressive surgery from 15 hospitals in 10 different countries between December 2011 and December 2019. We analyzed the cumulative incidence of seizures within one year after decompressive surgery and performed an exploratory analysis of potential factors associated with the incidence of post-surgery seizures, adjusted for competing mortality risk.

**Results:**

Of 116 included patients (median age 38 years [IQR 27–46], 68% female), 26 (22%) had seizures within one year after decompressive surgery. The mortality-adjusted cumulative one-year incidence of a first seizure after surgery was 23% (95% CI 16–31). Only aphasia at presentation predicted post-surgery seizures. Among patients who had a post-surgery seizure, the first seizure occurred despite active treatment with anti-seizure medications in 83% of patients. Recurrent seizures within the first year after surgery were reported in 63% of patients.

**Discussion and conclusion:**

Despite extensive use of anti-seizure medications, in patients with cerebral venous thrombosis treated with decompressive surgery the rate of seizures was 23% (95% CI 16–31) within one year after surgery. Of the patients who had a seizure after surgery, 63% had a recurrent seizure within the first year post-surgery.

## Introduction

Decompressive surgery is recommended to prevent death in patients with severe CVT and impending brain herniation (1% of all patients with CVT). [1–3] The cumulative incidence of seizures among patients with CVT who undergo decompressive surgery is unknown as are potential predictors of seizures in this specific patient category. Known predictors of late seizures in CVT, such as status epilepticus in the acute phase and intracerebral hemorrhage, are thought to be mostly markers of severe CVT with parenchymal brain lesions. [4] These markers may not discriminate seizure risks among patients treated with decompressive surgery, who all have severe CVT. Yet, information on risk of seizures is important to improve patient education and identify patients at high risk of seizures who might benefit from intensive anti-seizure medication (ASM) treatment or be enrolled in trials on epileptogenesis prevention [5].

We therefore aimed to analyze the cumulative incidence of seizures within the first year after decompressive surgery for CVT, adjusted for competing risk of death, in a consecutive cohort of patients with CVT treated with decompressive surgery. In addition, we aimed to explore potential predictors of post-surgery seizures in this patient group.

## Patients and methods

### Study population, data collection, and definitions

We performed a sub-study of DECOMPRESS-2, a prospective, international, multicenter, observational cohort study performed in 15 different hospitals in 10 different countries [6]. Consecutive patients aged ≥ 16 years with cerebral venous thrombosis treated with decompressive surgery were enrolled from December 2011 to December 2019. Cerebral venous thrombosis had to be diagnosed on MRI, MR venography, CT venography, or digital subtraction angiography, in accordance with guidelines [1, 2]. CVT associated with head trauma, or another intracranial disease which might pose an additional indication for neurosurgery (e.g., large subdural hematoma, ruptured arteriovenous malformation, dural arteriovenous fistula, or developmental venous anomaly), was an exclusion criterion. Decompressive surgery included hemicraniectomy, hematoma evacuation, or both.

Detailed information on demographics, CVT risk factors, symptoms and signs, brain imaging, surgery and other treatments, and outcome of patients (including any seizures) was collected with a standardized case report form at baseline and at two routine follow-up moments: 6 (± 1) and 12 (± 1) months after decompressive surgery. All data were crosschecked at the lead center, Hospital de Santa Maria in Lisbon, Portugal.

For the current sub-study, we retrospectively collected additional data with regard to date of first seizure post-decompressive surgery, seizure recurrence, and status epilepticus. Seizures and status epilepticus were defined as diagnosed by the local physician. Data on individual ASM use had been prospectively collected but was not a primary outcome of the DECOMPRESS-2 study. Current guidelines do not specifically address the use of postoperative prophylactic ASM after decompressive surgery for CVT, [2, 3, 7, 8] and policy with regard to prophylactic ASM therefore differed among participating centers as well as per patient within participating centers.

### Standard protocol approvals, registrations, and patient consents

This study was approved by the ethical committee of Hospital de Santa Maria in Lisbon, Portugal. Informed consent for the use of pseudonymized care data was obtained if required according to local laws and regulations.

### Statistical analysis

Information on seizures was missing in 2 patients, almost all study variables were missing in these patients and they were excluded from the analysis. We compared baseline characteristics of patients with and without one or more seizures until the last follow-up post-decompressive surgery (‘post-surgery seizures’). Dichotomous variables were compared using the chi-square test; continuous variables were all non-normally distributed and were compared with the Mann–Whitney *U* test. Comparisons were not adjusted for multiple testing and should be interpreted as exploratory. We estimated the cumulative incidence (with 95% confidence interval [95% CI]) of a first post-surgery seizure using a Fine–Gray model to adjust for competing mortality risk [9]. Patients who did not have a post-surgery seizure and did not die during the study period were censored at the time of last follow-up assessment (usually 12 months after the decompressive surgery). In addition, we analyzed potential predictors of post-surgery seizures. Predictor selection was based on results of the bivariate analyses and univariable Fine–Gray regression, with a maximum of 10 seizure events per variable to protect model stability. Univariable regression was performed for variables with a *p* value < 0.1 in bivariate analysis. Given the multicenter study design, with varying ASM policies across hospitals, combined with the expected small number of outcome events due to the limited sample size, we could not distinguish ASM treatment effects from center-level confounding and confounding by indication. Therefore, our model focused on patient-level neurological predictors rather than potential effects of treatment policies. Data were missing in 0–3% for co-variables included in the regression analysis. These were imputed using multiple imputation. Predictors that were statistically significant in univariable regression were included in the multivariable Fine–Gray regression analysis. The proportional hazards assumption was evaluated by graphical examination of the Schoenfeld residuals.

## Results

**Fig. 1:**
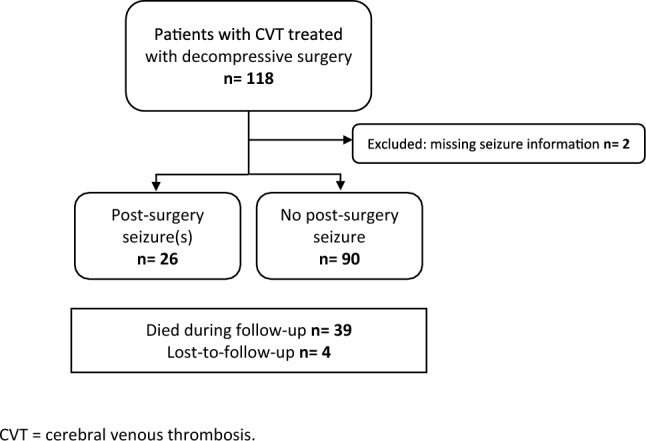
Flowchart of patient selection. *CVT* cerebral venous thrombosis

A total of 116 patients were included in the study. 26 patients had one or more seizures post-surgery, while 90 did not have post-surgery seizures (Fig. 1). A total of 39 patients died during the study follow-up period: 4 in the post-surgery seizure group and 35 in the no post-surgery seizure group. Four patients were lost-to-follow-up prior to the 12-month follow-up appointment. The median overall follow-up time including those who died was 350 days (interquartile range [IQR] 65–378).

Baseline characteristics among patients with and without post-surgery seizures are described in Table 1. Age and gender were comparable between the two groups. Patients who developed seizures post-surgery more commonly presented with aphasia and visual field defects at diagnosis compared to those who did not develop seizures post-surgery. In addition, they more often underwent surgical decompression and hematoma evacuation, rather than decompression only, compared to those who did not develop seizures post-surgery. There was no difference between the two groups with regard to the proportion of patients who had seizures prior to surgery (65% in the post-surgery seizure group versus 53% in the no post-surgery seizure group), status epilepticus prior to surgery, intracerebral hemorrhage at presentation or prior to surgery, subdural hematoma at presentation, or in the size of the largest parenchymal lesion at presentation or prior to surgery.

**Table 1:** Baseline characteristics

	Post-surgery seizure: *N* = 26 *n*/*N* (%)	No post-surgery seizure: *N* = 90 *n*/*N* (%)	*p* value
Demographics			
Female sex	18/26 (69)	61/90 (68)	1.00
Age—median (IQR)	40 (30–51)	38 (26–45)	0.94
CVT risk factors^a^			
Oral contraceptive use^b^	9/18 (50)	17/59 (29)	0.15
Other prothrombotic drug use	3/26 (12)^c^	8/88 (9)^d^	0.71
Any infection	2/26 (8)	8/88 (9)	1.00
Cancer	1/26 (4)	3/87 (3)	1.00
Puerperium^b^	2/18 (11)	14/59 (24)	0.33
Pregnancy^b^	0/18	6/59 (10)	0.33
Symptoms and signs from onset to CVT diagnosis^e^			
Headache	24/26 (92)	80/89 (90)	1.00
Motor deficit	19/26 (73)	55/89 (62)	0.36
Aphasia	11/26 (42)	17/90 (19)	0.02
Visual field defect	7/26 (27)	7/88 (8)	0.02
Coma^f^	8/26 (31)	30/90 (33)	1.00
Seizure	15/26 (58)	45/90 (50)	0.51
Brain imaging at CVT diagnosis^a^			
Parenchymal brain lesion	22/26 (85)	81/89 (91)	0.46
Intracerebral hemorrhage^g^	21/26 (81)	76/89 (85)	0.55
Non-hemorrhagic parenchymal lesion	6/26 (23)	16/89 (18)	0.58
Size of largest parenchymal lesion in cm—median (IQR)	7.0 (6.1–9.5)	7.7 (6.0–8.7)	0.93
Subdural hematoma	2/26 (8)	7/89 (8)	1.00
Subarachnoid hemorrhage	7/26 (27)	16/89 (18)	0.40
Last brain imaging prior to decompressive surgery^h^			
Intracerebral hemorrhage^g^	26/26 (100)	81/90 (90)	0.21
Size of largest parenchymal lesion in cm—median (IQR)	7.0 (6.0–9.4)	7.9 (6.1–8.9)	0.93
Seizure(s) from onset to decompressive surgery^e^			
Any seizure before surgery	17/26 (65)	48/90 (53)	0.37
Multiple seizures before surgery	8/24 (33)	35/88 (40)	0.64
Status epilepticus before surgery	1/24 (4)	13/88 (15)	0.30
Decompressive surgery			
Time from CVT diagnosis to surgery in days—median (IQR)	1 (0.5–2)	1 (0–3)	0.82
Type of surgery			0.03
Combined decompression and hematoma evacuation	11/26 (42)	23/90 (26)	
Decompression only	14/26 (54)	67/90 (74)	
Hematoma evacuation only	1/26 (4)	0/90	

^a^ Categories overlap; ^b^ percentage of women, ^c^ i.e., hormone replacement therapy (*n* = 1), asparagine and methotrexate (*n* = 1), unknown (*n* = 1); ^d^ i.e., hormone replacement therapy (*n* = 6), prednisolone and methotrexate (*n* = 1), tranexamic acid (*n* = 1), unknown (*n* = 1); ^e^ i.e., from onset of CVT symptoms; ^f^ i.e., Glasgow Coma Scale score < 9; ^g^ i.e., hemorrhagic infarction or intracerebral hematoma; ^h^ Brain imaging repeated after baseline prior to surgery in 64% of patients. *IQR* interquartile range; *CVT* cerebral venous thrombosis

Two patients had a post-surgery seizure within two weeks of cranioplasty. No post-surgery seizures were associated with any central nervous system infection or intracranial bleeding complication during follow-up.

**Fig. 2:**
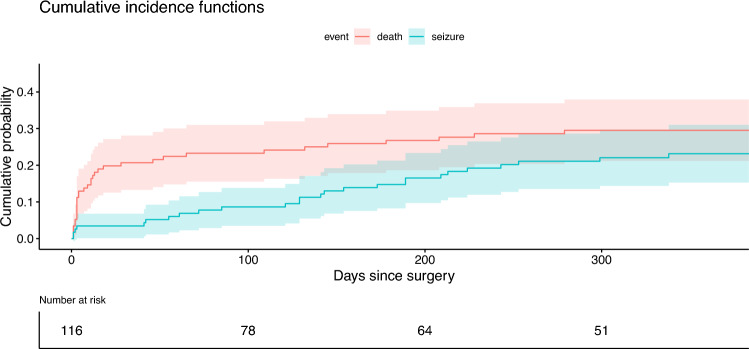
Cumulative probability of seizures within 1 year of decompressive surgery for CVT adjusted for competing mortality risk. Estimated cumulative probability of a first seizure (blue line) adjusted for cumulative probability of death (red line). Bands represent the 95% confidence intervals. *CVT* cerebral venous thrombosis

Figure 2 represents the cumulative incidence of first seizures during the first year after decompressive surgery for CVT, adjusted for competing risk of death. The mortality-adjusted cumulative one-year incidence of first post-surgery seizures was 23% (95% CI 16–31). Median time to first post-surgery seizure was 141 days (IQR 55–213; i.e., 4.6 months). Recurrent seizures within the first year after surgery were reported in 15/24 (63%) patients. Status epilepticus was reported in 1/21 (5%) patient.

Table 2 describes the use of ASM at different time points during the study among patients who did and did not have pre- and post-surgery seizures. There were no patients with post-surgery seizures who never used any ASM during the study period. In 20/24 (83%) patients, the first seizure after surgery was reported to have occurred despite active treatment with ASM, namely in 14/15 (93%) patients who had had a pre-surgery seizure and 6/9 (67%) patients who had not had a pre-surgery seizure.

**Table 2:** Overview of anti-seizure medication use during the study period

	Post-surgery seizure: *N* = 26 *n*/*N* (%)			No post-surgery seizure: *N* = 90 *n*/*N* (%)		
	Total	Presurgery seizure *N* = 17	No pre-surgery seizure *N* = 9	Total	Presurgery seizure *N* = 48	No pre-surgery seizure *N* = 42
ASM before or on day of surgery	19/25 (76)	14/16 (88)	5/9 (56)	57/90 (63)	39/48 (81)	18/42 (43)
ASM during initial hospital admission	21/24 (88)	14/15 (93)	7/9 (78)	69/69(100)	45/45(100)	24/24 (100)
ASM use at the 6 month follow-up	20/21 (95)	13/13 (100)	7/8 (88)	44/60 (73)	27/32 (84)	17/28 (61)
ASM use at the 12-month follow-up	20/21 (95)	12/12 (100)	8/9 (89)	40/53 (76)	23/26 (89)	17/27 (63)
ASM at time of first post-surgery seizure	20/24 (83)	14/15 (93)	6/9 (67)	NA	NA	NA
Never used any ASM	0/25 (0)	0/16	0/9	19/90 (21)	3/48 (6)	16/42 (38)

*ASM* anti-seizure medication; *NA* not applicable

Among the 51 patients who did not have a pre-surgery seizure, 23 (45%) were given prophylactic ASM before or on the day of surgery, of whom 5 (22%) had a post-surgery seizure. Conversely, 28 (55%) patients without a pre-surgery seizure did not start ASM before or during surgery, of whom 4 (14%) had a post-surgery seizure.

Results of the Fine–Gray regression are in Table 3. ‘Aphasia at presentation’, ‘visual field defect at presentation’, and ‘combined decompression and hematoma evacuation surgery’ were selected for univariable regression based on a *p* value of < 0.1 in the bivariate analysis. Visual inspection of the Schoenfeld residuals did not demonstrate major deviations from the proportional hazards assumption. Based on the univariable regression results, ‘aphasia at presentation’ and ‘visual field defect at presentation’ were selected as potential predictors for multivariable Fine–Gray regression. In multivariable regression analysis, only ‘aphasia at presentation’ predicted post-surgery seizures.

**Table 3:** Potential baseline predictors of post-surgery seizures, adjusted for competing mortality risk

Potential predictor	Univariable sHR (95% CI)	Multivariable sHR (95% CI)
Aphasia at presentation	3.1 (1.4–6.6)	2.8 (1.2–6.2)
Visual field defect at presentation	2.8 (1.2–6.7)	2.3 (0.8–6.3)
Combined decompression and hematoma evacuation surgery	2.0 (0.9–4.3)	–

*sHR* sub-distribution hazard ratio; *95% CI* 95% confidence interval; *ASM* anti-seizure medication

## Discussion

In this international cohort study, the cumulative one-year incidence of a first seizure after decompressive surgery for CVT was 23% (95% CI 16–31). In 83% of patients who had a post-surgery seizure, the seizure occurred despite active treatment with ASM, and 63% of patients had a recurrent seizure within the first year after surgery. Aphasia at presentation predicted post-surgery seizures in multivariable analysis adjusted for competing mortality risk.

In a previous study on late seizures after CVT, approximately 7% of all patients with CVT had a late seizure within one year of CVT diagnosis [10]. Among the 45 patients treated with decompressive surgery in this previous study, 38% had a late seizure during the entire study period, but the duration of follow-up and mortality rate in this subgroup was not reported. In the current study, intracerebral hemorrhage at presentation or prior to surgery, subdural hematoma at presentation, coma at presentation or prior to surgery, seizures before surgery, and status epilepticus before surgery did not predict post-surgery seizures although these or similar predictors did predict late seizures among the entire patient population with CVT in a previous meta-analysis and large cohort study [4, 10]. This finding may support the hypothesis that predictors of late seizures in CVT in general are often markers of severe CVT, and therefore do not differentiate seizure risks among patients with CVT who undergo decompressive surgery, who all have severe CVT with parenchymal brain lesions.

Overall, late seizures have been found to be more common after CVT than following ischemic stroke [10, 11]. However, in previous retrospective cohort studies including 36 to 80 patients with malignant MCA infarction who underwent decompressive surgery, about half of all patients had seizures during a median follow-up time of 1 to 3 years [12–14]. The lower rate of ASM use in these studies compared to ours may explain this higher seizure risk. In addition, potential differences in surgical techniques, the retrospective study designs and the fact that competing mortality risk was not accounted for may have further contributed to this difference. As in the present study, few baseline differences were found between patients with decompressive surgery for MCA stroke who did and did not develop seizures after surgery: male sex was associated with seizures in one study, [13] and delayed surgery (> 42 h after stroke onset) was associated with seizures in another [12].

In the present study, only aphasia at presentation predicted post-surgery seizures in multivariable analysis. Aphasia commonly results from cortical lesions, which are also more often epileptogenic [15]. Unfortunately, detailed information on lesion location was not available in the current study. Further studies are needed to independently validate whether aphasia is truly associated with an increased incidence of seizures after decompressive surgery for CVT, and whether lesion location might actually be a more precise predictor of post-surgery seizures.

Identifying patients at high risk of post-surgery seizures may help us to more adequately inform patients about their risk of having a seizure and inform ASM treatment decisions. The importance of improved patient education is elicited by a study in which 15 patients with seizures after decompressive surgery for malignant MCA infarction were interviewed [13]. Only 4 (27%) of these patients had been informed prior to the seizure that they might have a seizure, and all patients indicated that they would have wanted to be informed beforehand if research showed that they were at high risk to have a seizure.

Determining which patients have an increased seizure risk may also help to improve preventive treatment strategies. In this study, most patients who had post-surgery seizures were actively treated with ASM at the time of the seizure. However, we did not have access to detailed information on ASM type, dose, therapy compliance, or serum concentrations. Furthermore, DECOMPRESS-2 was not designed to evaluate the potential benefit or harm of prophylactic ASM after decompressive surgery for severe CVT. Further studies are warranted to investigate the effectiveness of different ASM treatment strategies, and potential options for optimizing prevention and seizure control after decompressive surgery for severe CVT.

This large international cohort study analyzed the incidence of seizures after decompressive surgery for CVT, and explored potential predictors of post-surgery seizures in this patient group. Several limitations of the study should be taken into account. First, the sample size of this study was small, leading to relatively imprecise estimates and limiting our analysis of potential predictors of post-surgery seizures. Nevertheless, our study represents the largest prospective cohort of patients with CVT treated with decompressive surgery to date. Second, data on seizure recurrence and status epilepticus were collected retrospectively. Third, diagnoses of CVT, seizures, and status epilepticus were not centrally adjudicated but diagnosed by the local physician. Fourth, most patients in this study were treated with ASM; thus, our study results should be interpreted as representing ‘real-world data’ rather than the natural course of post-surgery seizures in this patient group. Fifth, as mentioned above, detailed information on parenchymal lesion location was not available in the current study.

## Conclusion

Despite extensive use of anti-seizure medications, the cumulative one-year incidence of a first seizure after decompressive surgery for CVT was approximately 23% (95% CI 16–31). Of the patients who had a post-surgery seizure, 63% had a recurrent seizure within the first year after surgery.

## Data Availability

Anonymized data will be made available upon reasonable request by qualified researchers approved by the DECOMPRESS-2 Steering Committee.
